# Cadmium triggers an integrated reprogramming of the metabolism of *Synechocystis *PCC6803, under the control of the Slr1738 regulator

**DOI:** 10.1186/1471-2164-8-350

**Published:** 2007-10-02

**Authors:** Laetitia Houot, Martin Floutier, Benoit Marteyn, Magali Michaut, Antoine Picciocchi, Pierre Legrain, Jean-Christophe Aude, Corinne Cassier-Chauvat, Franck Chauvat

**Affiliations:** 1 Commissariat à l'Energie Atomique, Institut de Biologie et Tecnologies de Saclay, Service de Biologie Intégrative et Génétique Moléculaire, CEA Saclay F-91191 Gif sur Yvette CEDEX, France; 2 Centre National de la Recherche Scientifique, Unité de Recherche Associée 2096 CEA Saclay, F-91191 Gif sur Yvette CEDEX, France

## Abstract

**Background:**

Cadmium is a persistent pollutant that threatens most biological organisms, including cyanobacteria that support a large part of the biosphere. Using a multifaceted approach, we have investigated the global responses to Cd and other relevant stresses (H_2_O_2 _and Fe) in the model cyanobacterium *Synechocystis *PCC6803.

**Results:**

We found that cells respond to the Cd stress in a two main temporal phases process. In the "early" phase cells mainly limit Cd entry through the negative and positive regulation of numerous genes operating in metal uptake and export, respectively. As time proceeds, the number of responsive genes increases. In this "massive" phase, Cd downregulates most genes operating in (i) photosynthesis (PS) that normally provides ATP and NADPH; (ii) assimilation of carbon, nitrogen and sulfur that requires ATP and NAD(P)H; and (iii) translation machinery, a major consumer of ATP and nutrients. Simultaneously, many genes are upregulated, such as those involved in Fe acquisition, stress tolerance, and protein degradation (crucial to nutrients recycling). The most striking common effect of Cd and H_2_O_2 _is the disturbance of both light tolerance and Fe homeostasis, which appeared to be interdependent. Our results indicate that cells challenged with H_2_O_2 _or Cd use different strategies for the same purpose of supplying Fe atoms to Fe-requiring metalloenzymes and the SUF machinery, which synthesizes or repairs Fe-S centers. Cd-stressed cells preferentially breakdown their Fe-rich PS machinery, whereas H_2_O_2_-challenged cells preferentially accelerate the intake of Fe atoms from the medium.

**Conclusion:**

We view the responses to Cd as an integrated "Yin Yang" reprogramming of the whole metabolism, we found to be controlled by the Slr1738 regulator. As the Yin process, the ATP- and nutrients-sparing downregulation of anabolism limits the poisoning incorporation of Cd into metalloenzymes. As the compensatory Yang process, the PS breakdown liberates nutrient assimilates for the synthesis of Cd-tolerance proteins, among which we found the Slr0946 arsenate reductase enzyme.

## Background

Photosynthetic organisms that support much of the life on Earth, in using solar energy to renew the oxygenic atmosphere and make up organic assimilates essential to the food chain [1,2] are frequently challenged with toxic reactive oxygen species (ROS) generated by respiration and photosynthesis [3], and toxic metals that constitute persistent pollutants because they cannot be degraded. One of them, Cadmium (Cd), is very abundant in the environment as it is often combined with sulfur in Earth's crust, and it is also intensively spread out as (i) a by-product of zinc mining, (ii) the burning of fossil fuel, (iii) the dispersal of sewage sludge and phosphate fertilizers, and (vi) the manufacturing of paints, batteries and screens [4]. Subsequently, Cd can be transferred to the food chain, and bio-accumulated in human where it has a half-life greater than 20 years [5] and causes various diseases by as yet unclear processes [6]. Even metals that are essential to enzyme activity, such as zinc and iron [7,8], can become toxic when occurring in excess. This toxicity is likely due to the poisoning replacement of the cognate metal cofactor of diverse metalloenzymes, a phenomenon sometimes leading to oxidative stress [9].

Cyanobacteria, the most abundant photosynthetic organisms on Earth [10], are attractive models to investigate the interrelations between metal toxicity and oxidative stress, because they perform the two metal-requiring [8] ROS-generating processes [3], photosynthesis and respiration, in the same membrane system [11]. Furthermore, cyanobacteria share a wide range of genes in common with plants [12], in agreement with they being the likely ancestor of chloroplast [13]. Thus, lessons learned from stress responses in cyanobacteria will also greatly facilitate the understanding of how plant cells face environmental challenges. This is important, as Cd has been reported to be toxic to plants by as yet unknown processes that may [14] or may not [15] impair photosynthesis. Moreover, cyanobacteria are also suitable for biosensor and/or bioremediation applications [16,17].

Using the model cyanobacterium *Synechocystis *PCC6803 that possesses a small genome [18] fully sequenced, and easily manipulable with replicating plasmids [19-21], we have analyzed the global responses of photosynthetic cells challenged with Cd, H_2_O_2 _(the paradigm ROS agent) or drastic changes of availability of either Fe or Zn, through (i) DNA microarrays; (ii) absorption spectroscopy; (iii) oxygen evolution; (iv) Western blot; (v) targeted gene inactivation and (vi) assays of cell fitness. We show that Cd triggers a "Yin Yang" integrated reorganization of the cyanobacterial metabolism, under the control of the Slr1738 regulator. The "Yin" ATP-sparing downregulation of cell metabolism likely limits Cd uptake and poisoning incorporation in place of the cognate metal cofactor of metalloenzymes. The compensatory "Yang" breakdown of the photosynthetic machinery that impairs ATP production, liberates nutrient assimilates that become available for the synthesis of Cd-toxicity protecting enzymes, among which we found the Slr0946 arsenate reductase.

## Results

### Transcriptional regulations elicited by Cd are slower and more sustained than those triggered by H_2_O_2_

The transcriptome approach was used to characterize the kinetics of global changes in *Synechocystis *PCC6803 (*Synechocystis*) gene expression elicited by noxious agents, which were continuously applied to the cells to mimic the persistent character of stresses encountered in Nature. Exponentially growing cells were exposed to CdSO_4 _(50 μM) or H_2_O_2 _(3 mM) for increasing periods of time that triggered a wide range of changes in cellular viability (from 100% to less than 10%) and regulation (number of responsive genes and magnitude of changes in expression), as required for a thorough investigation of stress responses (Table 1). For each time point, total RNA were isolated from stressed and unstressed cells, reverse-transcribed, differentially labeled (dye swapped), hybridized together (stressed versus unstressed samples) and analyzed with DNA glass microarrays (two slides per each time point), as described in Methods. Our data (Table 1 and see Additional files 1, 2, 3, 4) showed that the Cd-elicited regulation could be divided in two main temporal phases. The early phase was moderate since only 151 genes responded to Cd during the first 60 min of treatment, and the changes were mostly up-regulation. The second phase occurring between 90 to 360 min of treatment was massive, with about 1,222 responsive genes equally distributed between up- and down-regulated genes. The relevance of the "early" and "massive" phases of Cd responses was verified by performing an independent biological repeat of both the time points 60 min (early phase) and 300 min (massive phase), and using an appropriate statistical test (Methods) to analyze all data (see Additional files 2, 3). Indeed, a large number of the Cd-regulated genes (791) appeared to be differentially expressed between the two temporal phases of responses.

**Table 1:** Influence of Stress on the Viability and Global Transcription Profile

**Treatment**	**Cadmium**										**Hydrogen peroxide**					**Fe depletion**		**Fe excess**		**Zn excess**	
*[C]*	*50 μM*										*3 mM*					*2-0*	*1-0*	*16 mM*		*776 μM*	
**Time (min)**	**15**	**30**	**60**	**75**	**90**	**180**	**300**	**300'**	**360**	**960**	**15**	**30**	**180**	**300**	**420**	**2,800**	**2,800**	**240**	**360**	**30**	**240**

% survival	100	98	90	88	85	70	57	59	43	<10	100	98	64	45	19	55	43	80	15	99	70
Induced genes	22	88	46	52	293	299	310	451	283	51	447	408	170	68	75	106	154	42	34	38	245
Repressed gene	8	17	10	13	250	315	328	439	310	26	490	478	92	12	55	104	109	98	100	22	221

Wild type cells were challenged for the indicated durations prior to survival and transcriptome analyses (Methods). In the case of the Fe starvation stress, the switch in Fe concentration is indicated as *1-0 *(standing for 1 μM then 0 μM) or *2-0 *(standing for 2 μM then 0 μM). The time point 300 min of the kinetic of Cd responses was repeated twice (independent biological repeats, columns 300 and 300') with the two different versions of the Cyanochip DNA microarrays, leading to very reproducible results (see Additional files 2 and 4).

Genes were considered differentially regulated whenever their level of expression was changed at least 1.9 fold.

By contrast, the transcriptional responses to H_2_O_2 _(3 mM) were faster and briefer than those to Cd (Table 1 and see Additional files 2, 3, 4). The massive phase of H_2_O_2_-mediated regulation encompassed the time points 15 min and 30 min (1,300 genes controlled, equally distributed between up- and down-regulation), while the late phase occurred between 180 min and 420 min (344 genes controlled, mostly positively), a time period in which most fast-responsive genes had returned to normal expression level (see Additional file 3).

### Cadmium antagonistically controls the genes operating in protein synthesis (downregulation), and protein maturation and degradation (upregulation)

Among the earliest responses to Cd (noticeable within the first 30 min of exposure) was the upregulation (see Additional file 4 panel A) of chaperones and proteases genes, the number of which increased during the massive phase of responses (after 60 min.). This regulation was accompanied with the downregulation of most ribosomal proteins genes (noticeable at 90 min, see Additional file 4 pannel A), and, comforting our data, we noticed that operonic genes were co-regulated. By contrast, most aminoacyl-tRNA synthetases genes were unaffected by Cd (see Additional files 2 and 6). Considering the normal level of expression [22] and the response to Cd (this study) of aminoacyl-tRNA synthetases genes (moderate expression, unresponsive to Cd) and ribosomal proteins genes (strong expression, turned down by Cd), we think that Cd-challenged cells preferentially downregulate those genes whose expression represents a metabolic burden. This interpretation is comforted by the findings that photosynthesis genes normally expressed to a high level [22] were also turned down by Cd (see below).

Similarly, H_2_O_2 _downregulated ribosomal protein genes (see Additional file 4, panel A), and did not affect aminoacyl-tRNA synthetase genes (see Additional file 3).

Also interestingly, we found that Zn excess partly mimics the Cd-mediated control of genes involved in protein folding and turnover (upregulation) or protein synthesis (downregulation), which were little affected by Fe availability (Table 1 and see Additional file 4).

### Cd and to a lesser extent H_2_O_2 _downregulate photosynthesis genes

**Figure 1:**
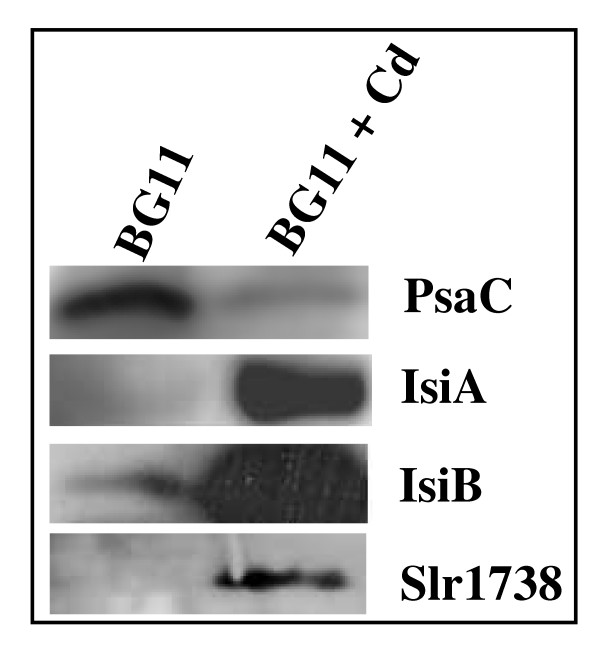
**Influence of cadmium on the abundance of selected proteins**. Cells were incubated for the indicated durations on solid media with or without (0 for untreated control) CdSO_4 _(50 μM, 360 min.) prior to disruption. 5 μg of crude cell extracts were analyzed by Western blottings (Methods), using the antibodies directed against the indicated proteins.

A very important target of Cd toxicity was the photosynthesis (PS) machinery that uses several electron-transfer complexes, the photosystemII (PSII) and its phycobilisomes (PBS) antennae, the cytochrome b6/f, the photosystemI (PSI) and the ATPase, to produce ATP and NADPH [8] required for the assimilation of inorganic nutrients. In addition, the PS activity can also generate toxic reactive oxygen species (ROS) in case of light excess [23]. Most of these PS genes were downregulated after 75–90 min. of Cd challenge (see Additional file 4 pannel B). The validity of these data was substantiated by the observed co-regulation of the following operons *psaAB*, *psbCD1*, *apcABC *and *atpIHGFDACBE*. In addition, we also observed the well-known [24] antagonistic iron regulation of the ssl0020 ferredoxin gene (repressed by Fe starvation) and *isiB *flavodoxin gene (induced by Fe limitation). Furthermore, we have verified the Cd-elicited downregulation of the *psaC *gene at the level of protein abundance (Fig. 1). Also consistent with the Cd-elicited downregulation of PS genes, we found (see Additional file 4 pannel B) that Cd (i) repressed most pigment synthesis genes, namely: *hemA*, *hemL*, *hemB*, *hemE*, *hemF*, *hemN*, *chlN*, *chlB*, *chlL*, *por*, *ho1*, *ho2*, *cbiX*, *crtH*, *crtR*, *crtD*-homolog and *alg*-homolog, and (ii) induced the *nblA *operonic genes that operate in PBS degradation [25].

**Figure 2:**
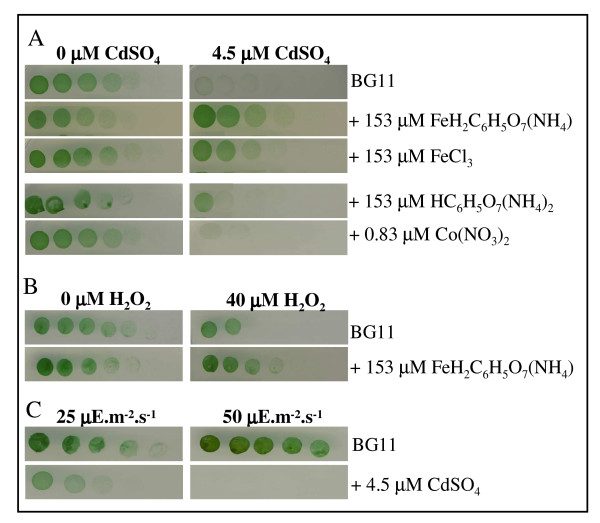
**Effect of metal, hydrogen peroxide and light fluence on cellular growth**. Four fold serial dilutions of mid log phase liquid cultures were spotted onto BG11 plates with or without the indicated agents, incubated for 4–5 days and scanned (Methods). Panel A: Typical influence of iron ((NH_4_)FeH_2_C_6_H_5_O_7 _or FeCl_3_), cobalt (Co(NO_3_)_2_) and citrate ((NH_4_)_2_H_2_C_6_H_5_O_7_) on the toxicity of cadmium (CdSO_4_). Panel B: Typical influence of iron ((NH_4_)FeH_2_C_6_H_5_O_7_) on the toxicity of hydrogen peroxide (H_2_O_2_). Panel C: Influence of the light fluence on the toxicity of CdSO_4_. These experiments were repeated three times.

The global downregulation of PS and pigment synthesis genes has been observed in cells challenged by a high light stress [26-28] and, very interestingly, we noticed that many of the high light-inducible genes [25,29,30] were also upregulated by Cd (see Additional file 4 panel B) namely: *hliB*, *hliC*, *isiA *and *nblA*. Collectively, these findings suggested that Cd-exposed cells become light sensitive, an interpretation we validated through growth assays (Fig. 2C).

The aspects of Cd toxicity resembling light stress are presumably due to oxidative stress since they could be elicited by H_2_O_2 _too (see Additional file 4 panel B). These common responses included the downregulation of all ATPase genes and several PBS genes (*apcA*, *apcB*, *apcC*, *apcE *and *apcF*), as well as the concomitant upregulation of many high light-inducible genes (*hliC*, *isiA *and *nblA*). Unsurprisingly, H_2_O_2 _mimicked high light stress that generates ROS [3] more efficiently than Cd. Indeed (see Additional file 4 panel B), H_2_O_2 _upregulated several high light-inducible genes unaffected by Cd, namely: *hliA*, *hliD*, *ctpA *and *ftsH *(slr0228 and slr1604) the protease genes involved in the high-light induced turnover of the D1 protein of PSII [31]. Also interestingly, many PS genes downregulated by Cd were actually upregulated by H_2_O_2_, namely: PSII (*psbB*, *psbJ*, *psbV *and *psbU*), PSI (*psaF*, *psaJ*, *psaD*, *psaI*, *psaM*) and PBS (*cpcC1*, *cpcC2 *and *cpcD*) (see Additional file 4 panel B).

In agreement with the upregulation of many genes induced by high light that triggers oxidative stress, we found (see Additional file 4 panel D) that Cd and/or H_2_O_2 _upregulated many anti-oxidant genes encoding thioredoxin reductase (*trxR*), thioredoxins (*trxA*, *trxM*), glutathione peroxidase (*gpx*), glutaredoxins (*grx*), glyoxalases (*glo*) and peroxidases (*gpx *and *ahpc*).

Similarly to Cd, Zn downregulated numerous PS genes (PBS, PSII, PSI and pigment synthesis, but not ATPase genes), and upregulated genes involved in protein turnover and tolerance to light/oxidative stress (see Additional file 4 panel B). By contrast, Fe controlled a few PS genes.

Also interestingly, the differential regulation of the cytochrome b6/f genes (TableS4B), encoding the predominant (*petC1*) or accessory (*petC2 *and *petC3*) Rieske iron-sulfur proteins [32], strongly suggests that alternative b6/f complexes are synthesized in response to changing environmental conditions.

### Spectroscopic confirmation that Cd elicits a more intense decline of the photosynthetic machinery than H_2_O_2_

**Figure 3:**
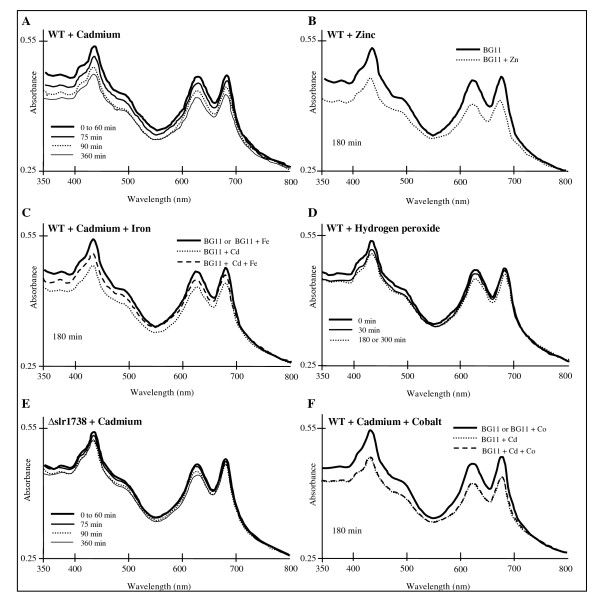
**Influence of metals and H_2_O_2 _on the abundance of photosynthetic pigments**. Typical absorption spectra of the wild type (WT) or slr1738 null-mutant (Δslr1738) cells following incubation for the indicated durations on solid BG11 medium with or without H_2_O_2 _(3 mM), CdSO_4 _(50 μM), Co(NO_3_)_2 _(350 μM), (NH_4_)FeH_2_C_6_H_5_O_7 _(350 μM) or ZnSO_4 _(350 μM or 776 μM). The spectra (normalized to light scattering at 800 nm) are displayed in panels A to F. These experiments were repeated three to five times.

That Cd- and Zn-excess turned down most photosynthesis genes and simultaneously upregulated protein degradation genes, suggested to us that these stresses decrease the abundance of the PS machinery. By comparison, we anticipated H_2_O_2 _to elicit a lower decline of the PS apparatus, in downregulating a smaller number of PS genes (see above). These predictions were all validated by another global method i.e. absorption-spectroscopy, which showed that the cellular content of colored PS pigments was decreased strongly in response to Cd- and Zn-stresses (Fig. 3A and 3B) and weakly in response to H_2_O_2 _(Fig. 3D). As control experiments, we have verified that excess of Fe (with little influence PS-gene expression see above) or cobalt did not alter pigments content (Fig. 3C and 3F).

### Oxygen evolution confirmation that Cd impairs photosynthesis

To further demonstrate that Cd impairs photosynthesis we measured the rate of the whole photosynthetic electron transport (from H_2_O_2 _to CO_2_) of intact cells incubated with or without 50 μM Cd. As expected, the oxygen-evolving activity of Cd-treated cells was strongly decreased (2.5- and 7-fold after 3- and 6-h, respectively) as compared to that of untreated cells.

### Cd and H_2_O_2 _likely disturb metal homeostasis

Cd rapidly and continuously altered expression of numerous metal transport genes, indicating that it disturbs metal homeostasis (see Additional file 4 panel C). For instance, all members of the nine genes cluster involved in the tolerance to Ni (*nrsBACD *operon), Co (*coaRT *divergon, sll0794 and slr0797) and Zn (*ziaBR *operon and *ziaA *export ATPase) were upregulated by Cd. As one of the numerous findings attesting the relevance of our transcriptome data we observed (see Additional file 4 panel C) that Zn controlled the genes *znuA *(slr2043, Zn uptake, downregulation) and *ziaA *(Zn export, upregulation), as expected [24,33,34]. That Cd regulated both *znuA *(negatively) and *ziaA *(positively), whose product is homologous to the Cd-transporting ATPase CadA [35], suggesting that Cd might be transported via Zn transport systems. Cd also controlled the *corR*-*corT *divergon operating in Co efflux, as well as the *cbi *cluster and the *cbiX *gene involved in the biosynthesis of cobalamin the Co-dependent vitamin B12 [36]. These data suggest that Cd disturbs Co homeostasis and utilization.

A large part of the numerous genes (more than 20) dedicated to Fe acquisition (*feoB*, *fec*, *fhu *and *fut*) were found to be positively regulated by Fe starvation and turned down by Fe excess (see Additional file 4 pannel B), in agreement with previous Northern blot data [37]. Again, attesting the relevance of our data, we also observed the Fe starvation-mediated control (see Additional file 4 panel D) of the *isiAB *operon (upregulation) and the *fed1 *genes (downregulation), as expected [38].

H_2_O_2 _upregulated all Fe acquisition genes (see Additional file 4 pannel C), as well as (see Additional file 4 panel D) the *suf *genes involved in iron-sulfur cluster biogenesis [39]. These findings are reminiscent to what occurs in *E. coli *where oxidative-stressed cells induce Fe uptake and *suf *genes to accelerate the supply of Fe atoms for the reconstitution of damaged iron-sulfur clusters, in a process leaving no free Fe atoms available for the toxic Fenton chemistry [40-42]. Interestingly, Cd upregulated antioxidant and *suf *genes, as well as half the number of the Fe-uptake genes (see Additional file 4 panels C and D). These findings suggest that Cd damages Fe-S centers, and that the extra Fe atoms required for their repair might be provided not only by the presumably moderate increase in Fe uptake, but also by the breakdown of the Fe-rich photosynthetic machinery (Fig. 3A) that contains 21–23 iron atoms per PS unit [43].

### Iron availability controls the Cd-elicited decline of cell viability and PS machinery

The above-mentioned data led us to predict that Fe availability can influence not only cell tolerance to H_2_O_2 _and Cd, but also the Cd-elicited decline of the PS machinery. As anticipated, we found that the addition of Fe in the medium at the onset of the stresses increased cell resistance to H_2_O_2 _and Cd (Fig. 2A and 2B), and prevented the Cd-elicited decline of the PS machinery (Fig. 3C). As a negative control, we verified that cobalt (Co) was unable to mimic these Fe-mediated protection effects (Fig. 2A and Fig. 3F).

### The Slr0946 arsenate reductase contributes to cadmium tolerance

**Figure 4:**
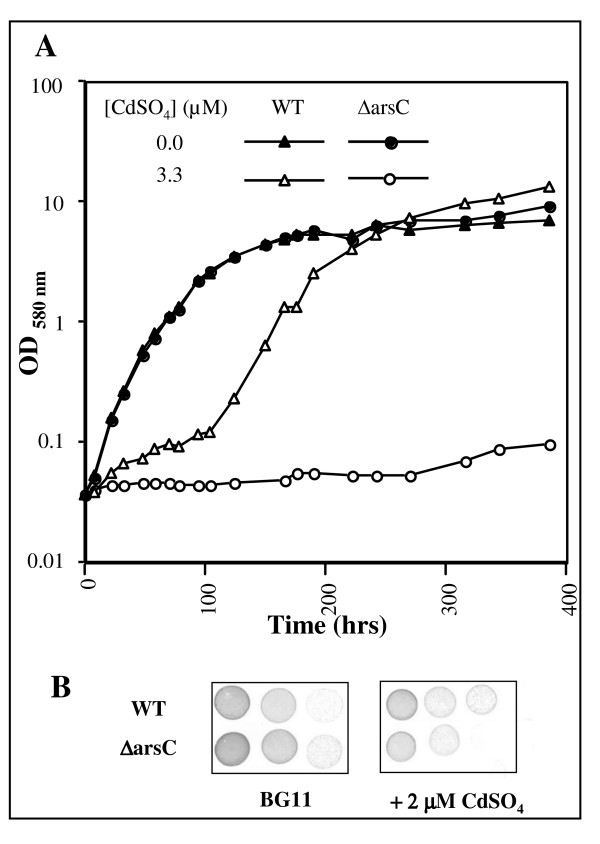
**Influence of cadmium on the growth of the wild type strain and *ΔarsC *mutant**. Typical growth of the wild type (WT, triangles) and *ΔarsC *mutant (circles) cultivated in liquid (PanelA) or solid (PanelB) BG11 media with or without CdSO_4 _(at the indicated concentration). These experiments were repeated three times.

We noticed that the *arsBHC *tricistronic operon (slr0944 to slr0946) operating in arsenic resistance [44,45] was rapidly and continuously upregulated by Cd (see Additional file 4 panel C). To confirm that the ArsC arsenate-reductase enzyme is a key factor in the tolerance to cadmium, we have deleted the *arsC *gene (Methods), and found the corresponding fully-viable *arsC *null mutant to be more sensitive to Cd than the WT strain (Fig. 4).

### Cd and H_2_O_2 _downregulate carbon metabolism genes, many of which encode ATP-requiring enzymes

Most CO_2 _concentrating mechanism (CCM) genes for the acquisition and assimilation of inorganic carbon (Ci) [46] were downregulated by Cd (see Additional file 4 pannel E), namely: (i) the *ndhF3*, *ndhD3 *and *cupA *tricistronic operon (CO_2 _uptake, NDH-I_3 _system); (ii) the *ndhF4 *and *ndhD4 *operon and the *cupB *gene (CO_2 _uptake, NDH-I_4 _system); (iii) the *cmpABCD *operon (HCO_3_^- ^transporter); (iv) the *sbtAB *operon (HCO_3_^- ^transporter); (v) the *cca *gene (carbonic anhydrase); (vi) the carboxysome genes *ccmK4 *and *ccmK-N *operon; (vii) the *prk *gene (phosphoribulokinase) and (viii) the *ppc *gene (phosphoenol pyruvate carboxylase). These results, together with the constitutive expression of the low-Ci inducible gene *ndhR *encoding the Ci-assimilation regulator [47], indicate that Cd challenged cells are not suffering from Ci starvation. Similarly, most carbon metabolism genes were turned down by Cd (see Additional file 4 panel E), namely: (i) *gpmA *(phosphoglycerate mutase); (ii) *eno *(enolase); (iii) *pyk2 *(pyruvatekinase); (iv) *pgk *(phosphoglycerate kinase); (v) *gap2 *(G3P-dehydrogenase); (vi) *glgC *(glucose-1-phosphateadenylyltransferase gene) involved in glycogen synthesis; (vii) *pgm *(phosphoglucomutase); (viii) *pfkA *(phosphofructokinaseI); (ix) *fbpI *(fructose1,6-biphosphataseI); (x) *fbaA *(fructose biphosphatealdolaseII); (xi) *pdhABCD *(pyruvate dehydrogenase); (xii) *icd *(isocitrate dehydrogenase).

Collectively, these data suggest that Cd downregulates citrate synthesis and conversion into 2-oxoglutarate that connects carbon and nitrogen assimilation pathways. This prediction was substantiated by the Cd-mediated downregulation of numerous genes operating in nitrogen metabolism (see below).

Very interestingly, we noticed that many of the carbon metabolism genes downregulated by Cd code for ATP-consuming enzymes such as *cmpABCD*, *fbpI*, *pgk*, *pfkA*, *prk *and *pyk1*. This negative regulation can be viewed as a part of a global ATP-sparing response to the Cd stress (See below).

Similarly to Cd, H_2_O_2 _downregulated many Ci acquisition genes (see Additional file 4 panel E): *ccm*, *cmp *and *sbt*, as well and numerous carbon metabolism genes: *pgk*, *gpmB *(slr1124 and slr1945), *eno*, *fbpI*, *carA*, *carB*, *pdhA*, *pdhB*, *pdhC *and *pdhD*. By contrast, Fe and Zn downregulated a few Ci acquisition genes such as *cmp *and *sbt *(Fe) and *ccm *(Zn), and had very little influence on carbon metabolism genes.

### Cd and H_2_O_2 _downregulate nitrogen metabolism genes, many of which encode ATP-consuming enzymes

Most genes for the acquisition and assimilation of nitrogen were negatively regulated by both Cd and H_2_O_2 _(see Additional file 4 panel F), namely: (i) *amt1 *and *amt2 *(ATP-requiring ammonium permease); (ii) *nrtABCD *operon (ATP-requiring uptake of nitrate); (iii) *urtABC *(ATP-requiring uptake of urea); (iv) *narB *(nitrate reductase); (v) *nirA *(nitrite reductase); (vi) *glnA *and *glnN *(the two ATP-requiring glutamine synthase); (vii) *murI *(peptidoglycan synthesis); (viii) *argB *(ATP-dependent N-acetylglutamate kinase for arginine synthesis), (ix) *cphA *(ATP-requiring [48] cyanophycin synthetase); (x) *hemA*, *hemF *and *hemL *(synthesis of PS pigments, see Additional file 4 panel B). Consistent with the downregulation of the glutamine synthase *glnA *gene we found that both Cd and H_2_O_2 _upregulate the *gifA *and *gifB *genes, which code for an inhibitor of GlnA activity [49]. In addition a few related genes were specifically downregulated by either Cd (*hemE *and *hemN*), or H_2_O_2_. The latter were the following: (i) *ureA *and *ureF *(urease); (ii) *carAB *(ATP-requiring carbamoyl phosphate synthase); (iii) *glsF *(ferredoxin-dependent glutamate synthase); (iv) *arG *(ATP-requiring argininosuccinate synthase for arginine synthesis); (v) *argH *(argininosuccinate lyase); (vi) *cphA *(ATP-requiring [48] cyanophycin synthetase); and (vii): *proA *(ATP-dependent gamma-glutamyl phosphate reductase for proline synthesis).

Together, our data strongly show that *Synechocystis *challenged with H_2_O_2 _or Cd downregulates numerous key genes encoding ATP-consuming enzymes involved in nitrogen acquisition and metabolisms. This finding is consistent with the above-mentioned negative regulation of ATP-requiring mechanisms for carbon assimilation and metabolism, and global protein synthesis (See above). We view these downregulations as an ATP-sparing process aimed at compensating the decline in ATP production caused by the negative regulation of ATPase and photosynthesis genes.

Fe (but not Zn) regulated numerous N acquisition and assimilation genes, suggesting that Fe homeostasis and nitrogen assimilation are intrinsically connected.

### Cd and H_2_O_2 _downregulate the two sulfur assimilation genes encoding ATP-dependent enzymes

Very interestingly, the genes *met3 *(sulfate adenylyltransferase) and *cysC *(adenylylsulfate kinase) encoding the two ATP-requiring enzymes of the cysteine-synthesis pathway appeared to be downregulated by both Cd and H_2_O_2 _(see Additional files 2, 3, 4). These data substantiate the Cd- and H_2_O_2_-elicited downregulation of ATP-consuming metabolic enzymes mentioned above.

### Prominent role of the Slr1738 regulator in the transcriptional responses and survival to Cd

**Figure 5:**
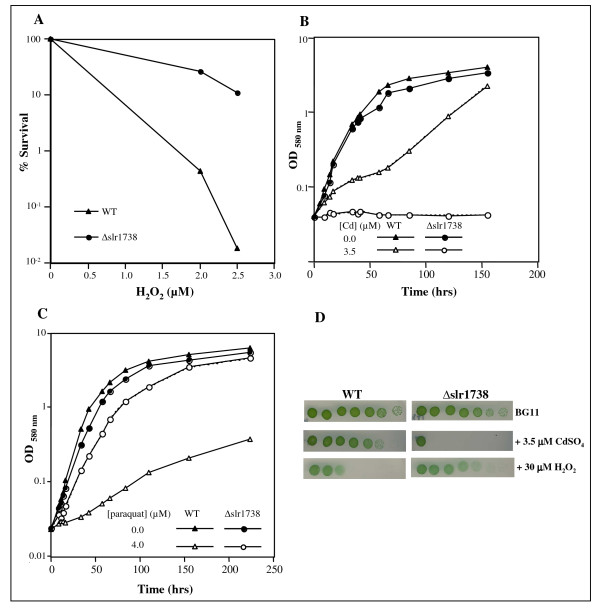
**Influence of the slr1738 gene on the tolerance to cadmium and oxidative stress**. Panel A: Typical survival to H_2_O_2 _of the wild type (WT, triangles) and Δslr1738 mutant (circles) cells. Panels B, C and D: typical growth of the WT and Δslr1738 cells cultivated in liquid (Panels B and C) or solid (Panel D) BG11 media with or without H_2_O_2_, paraquat and CdSO_4 _at the indicated concentration. These experiments were repeated three times.

To demonstrate that the Cd-elicited breakdown of the photosynthetic (PS) machinery (Fig. 3A) is a direct physiological response rather than a side effect of cell damage, we searched for a regulator controlling this breakdown process with the view that its inactivation in interfering with the PS decline should decrease the level of tolerance to Cd. Hence, we became interested in the slr1738 transcription regulator gene because it is upregulated by Cd (Fig. 1 and see Additional file 4 panel C), a finding which suggests that Slr1738 might be involved in the responses to Cd. We have deleted the slr1738 gene (see Methods) and found the corresponding slr1738 null-mutant (Δslr1738) to be fully viable in standard growth conditions (Fig. 5), in agreement with the small number of genes with an altered level of expression (23, data not shown). As expected, the Δslr1738 mutant was found to be less resistant to Cd than the WT strain (Fig. 5B and 5D), indicating that Slr1738 mediates some of the Cd-elicited regulations. To characterize the Slr1738-mediated responses to Cd, we used DNA microarrays to identify the genes whose transcript abundance in Cd-treated cells differed at least twofold between the Δslr1738 mutant and the WT strain. As expected, we found that the Cd-elicited downregulation of PS genes (PSII large subunits, PBS, pigments synthesis, ATPases, cytochrome b6/f complex) and the simultaneous upregulation of the *nblA *genes operating in phycobilisomes (PBS) degradation were all impaired in the slr1738 null mutant (see Additional file 4 panel B). These findings were confirmed through absorption-spectroscopy analyses of the cellular content of PS proteins. As expected, the Cd-elicited decline of pigmented proteins was truly lower in the Δslr1738 mutant than in the WT strain (compare Fig. 3E with Fig. 3A). The Cd-elicited downregulation of the genes coding for ribosomal proteins was also impaired in the Δslr1738 mutant (see Additional file 4 panel B). Slr1738 was also found to contribute to the Cd-mediated upregulation of the *suf *genes involved in Fe-S cluster assembly and repair (see Additional file 4 panels C and D), and the *ars *genes operating in tolerance to arsenic and cadmium (Fig. 4).

By contrast, Slr1738 is likely not involved in the Cd-mediated regulation of carbon metabolism (see Additional file 4 panel E), indicating that other Cd response regulator(s) remain to be identified.

Finally, the Δslr1738 mutant appeared to be more resistant to H_2_O_2 _and paraquat than the WT strain (Fig. 5A and 5C). This phenotype, unnoticed by previous workers [50,51], is consistent with the increased level of expression of various antioxidant genes (see Additional file 4) such as the sll1621 peroxiredoxin gene [50-52] and the sequence homology between Slr1738 and the *B. subtilis *PerR (peroxide) regulator whose inactivation increases the resistance to oxidative stress [53].

## Discussion

Photosynthetic organisms that support much of the biosphere are increasingly challenged by heavy metals, which are persistent in the environment since they cannot be degraded. Using the model cyanobacterium *Synechocystis *PCC6803 as the host, we have performed a thorough multidisciplinary analysis of the global responses of photosynthetic cells to cadmium (Cd), as well as to hydrogen peroxide (H_2_O_2_) and noxious concentrations of the essential metals iron and zinc because the disturbance of metal homeostasis can generate oxidative stress [9]. The presently reported data on the global responses to both the Cd and Zn stresses are novel. In the case of the H_2_O_2 _and Fe stresses our data confirmed and extended those obtained previously after less-extensive investigations. In the case of the H_2_O_2 _stress, the novelty of our report is that our temporal analysis made it possible to discriminate between early and late responses, unlike the previously performed single time-point analysis [51]. Concerning the Fe-starvation stress, we choose to study the responses to a continuous Fe limitation because stresses occurring in Nature are durable, whereas Singh and co-workers studied cells recovering from a transient Fe deficiency [38]. This presumably explains the fact that we observed the induction of a larger number of Fe acquisition genes than the previous workers. Furthermore, unlike previous workers we also studied the response to Fe excess to identify those genes oppositely responding to excess and deficiency of Fe, which are therefore likely responding to Fe *per se *rather than to another indirect stress signal.

The presently reported occurrence of two temporal phases in the responses to Cd and H_2_O_2_, with a stress-specific timing, emphasizes on the value of kinetic analyses of stress responses especially when they are to be compared. Indeed, one cannot assume that two stresses of equal duration and toxicity have the same biological effects, in term of gene regulation. This important fact is illustrated by our findings that cells challenged with Cd (50 μM) or H_2_O_2 _(3 mM) for the same period of time (i.e. 30 min.) leading to equal lethality (2%) displayed massive responses to H_2_O_2 _but only moderate (early) responses to Cd in term of the number of the genes regulated (see Additional files 2, 3, 4).

The biological significance of the genome-wide transcriptional responses to all presently tested stresses (Table 1, and see Additional file 1, 2, 3, 4, 5, 6) was validated using relevant assays (see Figs. 1 to 5) performed with appropriate metal doses that varied with the number of cells to be treated, and/or attested by the following evidences. First, in every case all gene members of the same operon were found to be co-regulated. Second, all Fe- and Zn-acquisition genes responded the expected way to the availability of their cognate metal. Third, most of the dispersed genes encoding the protein-subunits of the same complex were found to be co-regulated, as observed for instance in the case of the photosynthesis (PS) genes (see Additional file 4 panel B). Fourth, in agreement with the Cd-mediated downregulation of most genes PS and the concomitant upregulation of protein-degradation genes (see Additional file 4 panel A), we showed that Cd decreases both the abundance (Fig. 3) and activity (See oxygen evolution data in the Results section) of the PS machinery. Fifth, consistent with the Cd-elicited induction of the arsenate reductase gene *arsC *(see Additional file 4 panel C) we showed that *arsC *operates in Cd tolerance (Fig. 4). Thus, the ArsC enzyme has a great biotechnological potential in contributing to the tolerance to two widespread persistent pollutants: arsenic and cadmium. Possibly the ArsC enzyme, which employed glutathione (GSH) and glutaredoxin as reductants [45], could somehow "sequester" Cd in generating an hypothetical GSH-Cd complex less toxic than Cd. Sixth, as inferred from the Cd-elicited upregulation of numerous high light-inducible genes (see Additional file 4 panel A) we showed that Cd decreases the tolerance to light (Fig. 2C). Seventh, as anticipated from its Cd-elicited induction (Fig. 1 and see Additional file 4 panel C) we demonstrated that the Slr1738 regulator mediates several responses that are crucial to protection against Cd, such as the decline of the PS machinery and the upregulation of the *arsC *gene (Fig. 3, Fig. 4 and Fig. 5 and see Additional file 4).

The occurrence of large clusters of co-regulated genes, encoding ribosome, ATPase or Fe uptake proteins, suggests a mechanism of global control of gene expression involving chromosomal structure, similarly to chromatin remodeling in eukaryotic cells. This prediction is comforted by the findings (see Additional file 2) that the *Synechocystis *HU and Dps nucleoprotein genes, possibly involved in such structure-dependent global regulation [54], are regulated by Cd (see Additional file 4), positively (HU, sll1712) or negatively (Dps, slr1894).

Many of our data support the notion of metal selectivity. For examples, (i) Fe but not Zn mimicked the Cd-mediated downregulation of N acquisition and assimilation genes (see Additional file 4 panel F); and (ii) Zn but not Fe mimicked the Cd-mediated decline of the PS machinery (Fig. 3 and see Additional file 4) and the downregulation of ribosomal genes which is therefore not a general stress response. By contrast, numerous genes responded the same way to Cd, Fe, Zn and H_2_O_2 _(see Additional file 4), suggesting that reactive oxygen species might act in signal transduction of stress responses, as proposed [55].

Both H_2_O_2 _and Cd were found to upregulate numerous genes operating in tolerance to oxidative stress (see Additional file 4 panel D) and to the related high light stress (see Additional file 4 panel B) that also generates toxic reactive oxygen species (ROS) [23]. These findings, which were anticipated in the case of the H_2_O_2 _stress, were confirmed by showing that both H_2_O_2 _and Cd render cells light sensitive (Fig. 2C). In addition, both H_2_O_2 _and Cd upregulated the *suf *genes (see Additional file 4 panel D) involved in iron-sulfur cluster biogenesis or repair [39] and the Fe uptake genes (see Additional file 4 panel C; all genes in the case of H_2_O_2 _and half of them in the case of Cd). Consistently, we found that increasing the concentration of Fe in the medium increases the cell tolerance to both H_2_O_2 _and Cd (Fig. 2). These results are reminiscent to what occurs in oxidative-stressed *E. coli *cells [40-42]. They suggest that *Synechocystis *challenged with either H_2_O_2 _or Cd accelerates Fe uptake strongly (H_2_O_2_) or moderately (Cd) to provide extra Fe atoms for the repair of damaged Fe-S clusters. Having also noticed that Cd triggers a larger breakdown of the Fe-rich PS machinery than H_2_O_2 _(Fig. 3), we believe that cells challenged with H_2_O_2 _or Cd use two strategies to provide extra Fe atoms to the machinery that synthesizes or repairs Fe-S centers. H_2_O_2_-treated cells undergoing a limited PS-decline (Fig. 3D) mostly accelerate the intake of Fe from the medium, while Cd-stressed cells that moderately increase Fe intake breakdown a part of their abundant PS machinery (Fig. 3A), which normally contains 21–23 Fe atoms per PS unit [43]. Consequently, we predicted, and confirmed, that increasing the availability of Fe limits the Cd-elicited decline of the PS-machinery (Fig. 3).

Many of the key genes involved in acquisition and metabolism of C, N and S that were downregulated by Cd and H_2_O_2 _are coding for ATP-consuming enzymes (see Additional file 4 panels E and F). These responses can be viewed as an ATP-sparing process used by the cells to compensate for the decreased production of ATP caused by the decline of the PS apparatus (Cd and to a lesser extent H_2_O_2_) or of the respiration machinery (H_2_O_2 _but not Cd downregulates cytochrome oxidase genes, See TableS4B). Similarly, the downregulation of ribosomal genes (see Additional file 4 panel A) encoding normally abundant proteins [22] triggered by cells facing Cd and H_2_O_2 _is likely aimed at sparing C, N and S nutrients to compensate for the downregulation of the corresponding acquisition and assimilation genes.

Based on the presently reported findings, we view the responses to a continuous Cd stress as a two temporal-phases process. In the early phase occurring during the first 60 min of exposure (Table 1), Cd-stressed cells regulate mainly the genes operating in metal transport (see Additional file 4 panel C) and protein maturation and degradation (see Additional file 4 panel A). These responses presumably limit Cd entry into the cells and incorporation in place of the cognate metal cofactor of metalloproteins. In prolonged exposures (after 60 min.), these regulations are conserved, and even amplified in term of the number of responsive processes, thereby defining the next phase designated as "massive" for this reason. At this stage, the responses to Cd can be viewed as an integrated "Yin Yang" reprogramming of the whole cellular metabolism. As the Yin process, most key genes operating in uptake and assimilation of inorganic nutrients (C, N and S) and protein synthesis are turned down. These responses allow (i) the sparing of both energy (ATP) and reducing power (NAD(P)H) normally consumed by nutrient assimilation and subsequent metabolism, and (ii) the limitation of the poisoning incorporation of Cd in metalloenzymes. As the compensatory Yang process, the PS breakage of the PS machinery, which decreases the production of both ATP and NADPH, liberates Fe and C, N and S nutrient assimilates that can be recycled into the synthesis of Cd-tolerance enzymes such as the ArsC arsenate reductase (Fig. 4) and, presumably, other Cd-inducible enzymes: Suf proteins (see above), flavodoxin (IsiB), ferredoxin (FedII), flavoproteins (Flv2 and Flv4), glutathione peroxidase (Gpx1), peroxiredoxin (Ahpc-like), thioredoxin (TrxA), hydrogenase subunits (HypA, D, E) and the ZiaA (the ATPase homologous to the cadmium export ATPase of other organisms). Furthermore, other new Cd-tolerance enzymes might be discovered in the future, among the product of the orphan genes which apperaed to be upregulated by Cd (see Additional files 1 and 3). We showed that the Cd-induced Slr1738 regulator (Fig. 1 and TableS4) plays a central role in the protection against Cd (Fig. 5) in mediating several of the important regulations, such as the breakage of the PS machinery, the downregulation of ribosomal genes, and the upregulation of the *arsC *arsenate reductase and *suf *genes.

## Conclusion

Using the cyanobacterium *Synechocystis *PCC6803 as a model organism, we analyzed the global responses of environmentally important cells to stresses triggered by Cd (an abundant persistant pollutant), H_2_O_2 _(the paradigm ROS agent), or drastic changes in Fe availability, which appeared to modulate the tolerance to Cd and H_2_O_2_. Our results indicate that cells challenged with H_2_O_2 _or Cd use different strategies for the same purpose of increasing the supply of Fe atoms to the synthesis and repair of Fe-requiring metalloenzymes. While H_2_O_2_-challenged cells preferentially accelerate Fe intake, Cd-stressed cells preferentially breakdown the Fe-rich PS machinery to liberate Fe atoms. We view the responses to Cd as an integrated "Yin Yang" metabolic reprogramming. As the "Yin" process, the ATP- and nutrients-sparing downregulation of anabolism limits the poisoning incorporation of Cd into metalloenzymes. As the compensatory "Yang" process, the PS breakdown liberates nutrient assimilates for the synthesis of Cd-tolerance proteins. We found that this reprogramming is mediated by the Slr1738 transcriptional regulator that also operates in oxidative stress tolerance, in agreement with its sequence homology with the peroxide resistance regulator of *Bacillus subtilis*. Further studies will be necessary to understand the influence of Cd and H_2_O_2 _on the activity of Slr1738.

## Methods

### Bacterial strains, growth and survival analyses

The unicellular cyanobacterium *Synechocystis *PCC6803 was grown as described [56] at 30°C in liquid BG11 medium enriched with Na_2_CO_3 _(3.78 mM, final concentration), under continuous white light of standard fluence (2,500 luxes, i.e. 31.25 μE.m^-2^.s^-1^). When required kanamycin 50–300 μg.ml^-1 ^was added to the cultures. For growth assays, cells grown three times up to mid log phase (OD_580 _0.5 units, i.e. 2.5 × 10^7 ^cells.ml^-1^) were inoculated into fresh BG11 medium with or without CdSO_4 _or paraquat (at the indicated concentration), and OD_580 _were measured at time intervals. For survival analysis, cells in mid log phase were incubated for 1 h with H_2_O_2 _(at the indicated concentration), washed twice with BG11, plated on solid BG11, and the colonies were counted after 5–7 days of incubation under standard conditions. The influence of various agents on the growth and survival on solid media was assayed as follows. Four fold serial dilutions of liquid cultures were spotted as 15 μl dots onto BG11 plates with or without the indicated concentration of the tested agents. Plates were then incubated for 4–5 days under standard conditions prior to scanning. For survival analysis, cells were harvested, washed and resuspended in BG11 medium prior to plating and counting.

### Construction of knockout mutants of Slr0946 (arsenate reductase) and Slr1738 regulator

Specific oligonucleotides were used for PCR amplification from the *Synechocystis *genome [57] of each studied gene along with its two 0.3 kb-long flanking DNA segments that serve as platforms for homologous recombinations mediating targeted gene replacement [58]. The PCR products were independently cloned in the pGEMt plasmid (Pharmacia), and inactivated as follows. For slr0946, an internal 223 bp segment (starting from the 7^th ^nucleotide downstream of its GTG start codon) was substituted by the *Stu*I restriction site that was subsequently used to insert the *Hinc*II Km^r ^cassette originating from the pUC4K plasmid (Pharmacia). For slr1738 inactivation, the 388 bp segment beginning 6 nucleotides behind the ATG start codon was replaced by the *Sma*I site in which we cloned the *Hinc*II Km^r ^cassette. The resulting deletion cassettes slr0946::Km^r ^and slr1738::Km^r ^were sequenced (Big Dye kit, ABI Perking Elmer) prior to transformation to *Synechocystis*. Through PCR and sequence analyses, we verified that the antibiotic resistant marker had been inserted properly in the genome of the transformant clones, i.e. in place of the corresponding studied gene. These nullmutants grew healthy in standard conditions, demonstrating that both Slr0946 and Slr1738 proteins are dispensable to the viability of *Synechocystis*.

### Photosynthetic pigments determination by absorption spectrometry

Absorption spectra of whole cells grown or challenged on solid medium and resuspended in water, were monitored with a DU640 spectrophotometer (Beckman). Samples were adjusted for equal scattering at 800 nm. Carotenoids absorb light between 350 and 540 nm. The absorption maximum for phycocyanin is 630 nm and that for chlorophyll a are 442 nm and 681 nm. These experiments were repeated at least three times.

### RNA isolation

Because of their short half lives typical of prokaryotic transcripts, *Synechocystis *mRNA were rapidly prepared (in less than 2 min.) from cells grown or challenged on solid media as described [56]. Briefly, 300 ml of mid-log phase liquid cultures (2.5 × 10^7^cells.ml^-1^) grown in standard conditions were rapidly concentrated 40-fold by centrifugation and spotted as 20 μl dots on BG11 plates with, or without (control samples), CdSO_4 _(50 μM); (NH_4_)FeH_2_C_6_H_5_O_7 _(17 μM); ZnSO_4 _(776 μM); or H_2_O_2 _(3 mM). Then, plates were incubated for the indicated times (in min) prior to cell harvest and fast disruption with an Eaton press. Iron depletion analyses were performed with liquid cell suspensions to avoid uncontrolled liberation of Fe atoms from agar. Hence, exponentially growing cells washed in Fe-free medium were challenged for 48 h in liquid medium containing 0 to 2 μM of (NH_4_)FeH_2_C_6_H_5_O_7_. Then, cells were harvested by centrifugation and resuspension and disrupted. RNA were extracted [56] with the RNeasy kit from Qiagen (DNA microarrays kit) and treated with RNase-free DNase I (Roche). The RNA concentration and purity were determined by A260 and A280 measurement (A260/A280 > 1.9), as well as by migration on agarose gel to verify the absence of RNA degradation.

### DNA-microarray data acquisition and statistical analysis

The microarrays data presently reported have been deposited in the MIAME compliant NCBIs Gene Expression Omnibus [59] under the accession number GSE3755 (see Additional file 6) DNA microarrays (IntelliGene™ CyanoCHIP version 1.2 or 2.0, Takara), covering 2,891 (CyanoCHIP1.2) or 2,954 (CyanoCHIP2.0) of the 3,168 ORFs of *Synechocystis *were purchased from Cambrex Bio Science and manipulated as described [56]. Test RNA (from stressed cells) and corresponding control RNA (untreated cultures) were reverse transcribed, differentially labeled with Cy3 and Cy5 dyes and hybridized in a replicate dye swap. Arrays were immediately scanned with a GenePix™ 4000B scanner (Axon Instruments), and images were analyzed with GenePixTM Pro 4.0 (Molecular Devices). Spots were considered when they lack blemishes, deformations or dusts, and their fluorescence signal exceeded the local background plus 2 standard deviations. Then, signal intensity was determined by subtracting local background of each spot (GenePix™ Pro 4.0). Each GenePix Result file (.GPR) was converted to a TIGR Array viewer file (.TAV) using TIGR ExpressConverter version 1.7 for signal analysis. All spot intensities have been normalized with the LOWESS method [60], using the locfit function of the TIGR Midas version 2.19 [61] with the smooth parameter set to 0.33 as recommended [62]. Normalized measures served to compute the ratios of Cy3/Cy5 intensity and the associated log2-transform (denote log2-ratios) for each gene. For each replicated dye-swap, the average expression ratio of a given gene is calculated as the geometric mean of the two ratios [62].

Three lines of evidences attested the quality of our data. First our normalization method was validated with both internal (positive: *Synechocystis *DNA; negative: Salmon sperm DNA) and external (human TFR mRNA) controls. Second, for each dye-swap, correlation coefficients calculated between both replicates (see Additional file 5) appeared to be greater than 0.9 in most cases, thereby attesting the within-study reproducibility. Third, to analyze the within-platform variations, Cy3- and Cy5-labeled cDNAs were prepared from a single preparation of total RNA from unstressed cells, mixed together and hybridized to a microarray. The distribution of expression ratio ((see Additional files 4 and 6) showed that 90% of them fall within the range (0.80 – 1.25) and 99% within the range (0.64 – 1.57). Moreover, the mean of this distribution is equal to 1.00 (standard deviation equal to 0.13). Consequently, we felt confident to regard as regulated any particular gene the expression level of which was changed at least 1.9 fold.

### Identification of the two temporal phases of the responses to Cd and H_2_O_2_

We have considered each cDNA array as a split replicate, without averaging the dye-swap values. For each stress, the microarray data were dispatched in two groups each corresponding to a presumed kinetic phase. In the case of Cd the first group of data (15 mins to 60 mins) contains 8 replicates and the second (90 min to 360 min) contains 10 replicates, while for H_2_O_2 _the first group (15 min to 30 min) contains 4 replicates and the second (180 min to 420 min) contains 6 replicates. To identify genes with log2-ratios significantly different between the two time phases, p-values were first calculated for each gene using a moderated t-test based on an empirical Bayes analysis that is equivalent to shrinkage (or expansion) of the estimated sample variances towards a pooled estimate, resulting in a more stable inference. The p-values of the t-test were adjusted for multiple hypotheses testing, controlling the false discovery rate (FDR) as proposed by [63]. Thus, using a cut-off of the adjusted p-values at 0.05 gives and approximate level of False Discovery Rate (FDR) at 0.05. Using a strict cut-off of p = 1e-3 we found 791 genes differentially expressed in the two kinetic phases of responses to Cd (see Additional file 2) and 228 phase-responsive genes in the case of H_2_O_2 _(TableS3). The statistical analysis was carried out in the R language release 2.2, using the package limma [64] from the Bioconductor project [65].

### Measurement of photosynthetic activity

Cells incubated for 3 and 6 h on solid BG11 medium with or without CdSO4 (50 μM) were washed and resuspended in BG11 medium as described in the RNA isolation section. Photosynthetic oxygen-evolving activity of intact cells was measured at 30°C under saturating light intensity with a Clark-type oxygen electrode (Hansatech).

### Over-expression and purification of the Slr1738 protein fused to a hexahistidine tag

The Slr1738 coding sequence was PCR amplified from the *Synechocystis *genome, using appropriate oligonucleotide primers to embed its ATG initiation codon into a *Nde *I restriction site and introduce a *Bam *HI site behind its stop codon. The resulting *Nde *I-*Bam *HI restriction fragment was cloned into the pET28 (+) *E. coli *expression vector opened with the same enzymes, thereby allowing the in-frame fusion of the 6 × His tag with the Slr1738 amino acids sequence. After sequence verification (Big Dye kit, ABI Perking Elmer) the pET28-1738 plasmid was transformed into *E. coli *BL21 (DE3) selecting for resistance to kanamycin (50 μg.ml^-1^). Transformant cells were grown at 37°C in Km-containing Luria Bertani medium up to an optical density (A600) of 0.8. At that time, 1 mM isopropyl-thio-β-D-galactopyranoside (IPTG) was added to induce the synthesis of the 6 × His-Slr1738 protein, and cells were further incubated for 15 h at 30°C, harvested by centrifugation and resuspended in 20 ml of 20 mM Tris pH 8.0, 500 mM NaCl and 5 mM imidazole (lysis buffer). Cells were disrupted by sonication (Microson), centrifuged at 14,000 g for 20 min at 4°C, and the supernatant was applied to a nickel-nitrilotriacetic acid-agarose column (3 ml) equilibrated with 25 ml of lysis buffer. After washings with 30 ml of lysis buffer and buffer A (20 mM Tris pH 8.0, 500 mM NaCl and 50 mM imidazole), recombinant proteins were eluted with 6 ml of buffer B (20 mM Tris pH 8.0, 500 mM NaCl and 500 mM imidazole). 6His-Slr1738 containing fractions were pooled, desalted on a PD10 Sephadex G-25M column (Amersham Biosciences). The Purity of the 6His-Slr1738 protein was greater than 95%, as judged by SDS-PAGE electrophoresis.

### Western blot analysis of selected proteins

Crude cell extract (5 μg) of *Synechocystis *cells incubated on solid media with or without CdSO_4 _(50 μM, 360 min.) or H_2_O_2 _(3 mM, 30 min.) were harvested, disrupted (see above), electrophoresed on 13% SDS-PAGE [66] and transferred onto nitrocellulose sheets as described [67]. For detections we use the following rabbit antibodies: anti-Slr1738 (this work, dilution 1:20000); anti-psaC (dilution 1:1000) or anti-rbcL (dilution 1:5000) from Agrisera; anti-IsiA (kindly provided by Dr. A. Wilde, dilution 1:5000); anti-IsiB (kindly provided by Dr. M. Hagemann, dilution 1:5000). Horseradish peroxidase-conjugated goat anti-rabbit antibodies (dilution 1:4000) were used as second antibody, and immune complexes were revealed by chemiluminescence (ECL kit, Amersham Biosciences).

## Authors' contributions

LH participated in the design and realization of the transcriptome experiments in the wild type strain. MF constructed the slr1738 null mutant and carried out the transcriptome and phentotypic analysis of this strain. BM carried out the construction and phenotypic analyses of the *arsC*-null mutant, and participated to other cell-fitness analyses. MM participated in the bioinformatics analysis of the transcriptome data, and in the drafting of the relevant part of the manuscript. AP carried out the overproduction of Slr1738 protein and corresponding antibodies and performed the Western blot experiments. PL participated in the analysis and interpretation of the transcriptome data and in the drafting of the whole manuscript. JCA participated in the bioinformatics analysis of the transcriptome data, and in the drafting of the relevant part of the manuscript. CCC participated in the conception, acquisition, analysis and interpretation of all data; carried out the oxygen evolution measurements; and participated in the drafting of the whole manuscript. FC participated in conception and supervision of the whole study, and wrotes the manuscript. All authors read and approved the final manuscript.

## Supplementary Material

Additional file 1 Analysis of the unstressed versus unstressed control experiment. These file describe the distribution of allexpression ratio for the unstressed versus unstressed control experiment, showing the extreme, mean and quantile values. Click here for file

Additional file 2 Kinetic analysis of the transcriptional responses to cadmium. The data provided indicate for each gene (column 1), encoding the indicated protein (column 12), the log-ratio of response to the indicated duration of the cadmium treatment (columns 2 to 10) and the p-value obtained by the analysis (column 11). Click here for file

Additional file 3 Kinetic analysis of the transcriptional responses to hydrogen peroxide. The data provided indicate for each gene (column 1), encoding the indicated protein (column 8), the log-ratio of response to the indicated duration of the hydrogen peroxide treatment (columns 2 to 6), and the p-value obtained by the analysis (column 7). Click here for file

Additional file 4 Relevant List of Stress-Responsive Genes. The data provided indicate for each gene (extreme left and right columns), sorted by the physiological function they operate in, the log-ratio of response to the indicated duration of stresses. Click here for file

Additional file 5 Dye-Swap-correlations. The data provided indicate (for each time of the 5 kinetics) the correlation coefficient between the two ratio samples obtained with the two microarrays of each dye-swap. Click here for file

Additional file 6 GSM numbers of all microarray experiments. This table provides the links for accessing our microarray data which have been deposited in the GEO website. Click here for file
